# The rise of Bioscience in the East

**DOI:** 10.1186/1742-4690-7-106

**Published:** 2010-12-15

**Authors:** Kuan-Teh Jeang

**Affiliations:** 1 The National Institutes of Health, Bethesda, MD, USA

## Abstract

The rapid growth of bioscience in China is considered.

## 

A few days ago, the results of an international standardized test administered to students from 65 countries were released. The test known as the Program for International Student Assessment (PISA) was conducted by the Organization for Economic Cooperation and Development http://www.oecd.org/, a Paris based entity. The testing involved 5000 students per country of age 15 years and 2 months. The surprising results from the PISA were that students from China (Shanghai), who actually participated for the first time, performed uniformly better than their cohorts from other countries. PISA scores are scaled with the average being set at 500. As reported in the *New York Times *"On the math test, students in Shanghai scored 600, in Singapore 562, in Germany 513, and in the United States 487. In reading, Shanghai students scored 556, ahead of second-place Korea with 539. The United States scored 500 and came in 17th, putting it on par with students in the Netherlands, Belgium, Norway, Germany, France, the United Kingdom and several other countries. In science, Shanghai students scored 575. In second place was Finland, where the average score was 554. The United States scored 502 -- in 23rd place -- with a performance indistinguishable from Poland, Ireland, Norway, France and several other countries." Although there are many ways to interpret the results, at face value they speak to the impressive educational achievements of students in China.

As globalization trends toward economic parity in diverse regions, a few years ago I noted some interesting numbers. The statistics from the US National Science Foundation (NSF) showed that between 1995 and 2005 the output of worldwide science and engineering journal articles grew at an average annual rate of 2.3 percent, but the US growth rate was much lower, at an annual 0.6 percent, while the greatest increase in annual article productivity came from Asia: 6.6 percent. http://www.nsf.gov/statistics/seind08/. One might surmise that since 2005, growth in Asia (particularly China) is occurring at an even faster rate. In fact, the latest SCImago http://www.scimagojr.com scientific ranking of countries (based on data from year 2008) now lists China in second place with 231,000 published items, behind the United States (375,000 published items) and ahead of the third place United Kingdom (120,630 published items). And it is not surprising then, that in 2010 the Chinese economy for the first time surpassed the size of the Japanese economy, and China has eased into second place behind only the United States.

The experience at *Retrovirology *also verifies the rapidly changing China trend. For example, we surveyed papers published in *Retrovirology *over the last three year (2008, 2009, 2010) for those that come from China, Hong Kong and Taiwan. In 2008, the number of papers contributed from these locales was zero. By 2009, the number had increased to four [1-4]; and in 2010, there are five papers [5-9]. Indeed, the general uptrend in scientific productivity from China is reflected tangibly in papers being published in *Retrovirology*.

One could view the rapid development of bioscience in China as a challenge to traditionally held views of "American exceptionalism". On the other hand, one also must realize that diseases know no geographic boundaries, and solutions and cures developed in America are good for China and elsewhere; similarly breakthroughs in biology and medicine in China benefit not only the East, but also the West, the North, and the South. Apparently, it is in the latter spirit that non-Chinese scientists are more and more engaging their Chinese counterparts through scientific exchanges and attendance at the increasing numbers of meetings being held in China (see examples: http://meetings.cshl.edu/CSHAsia/index.html; http://www.scbameeting2011.org; http://www.meeting.edu.cn/webmedia/oemui/en/index.htm). One hopes that the rise of science in China presages a rising tide of scientific progress in many other developing countries. To the benefit of all, the United States, Europe, and other developed countries have important roles to play in fostering the quantity and quality of scientific excellence in the up-and-coming fledgling nations.
